# Tempforming Strengthening of a Low-Alloy Steel

**DOI:** 10.3390/ma15155241

**Published:** 2022-07-29

**Authors:** Anastasiia Dolzhenko, Rustam Kaibyshev, Andrey Belyakov

**Affiliations:** 1 Laboratory of Mechanical Properties of Nanostructured Materials and Superalloys, Belgorod State University, Belgorod 308015, Russia; dolzhenko_a@bsu.edu.ru; 2 Laboratory of Prospective Steels for Agricultural Machinery, Russian State Agrarian University—Moscow Timiryazev Agricultural Academy, Moscow 127550, Russia; kajbyshev@rgau-msha.ru

**Keywords:** low-alloy steel, tempforming, ultrafine lamellar microstructure, strengthening, impact toughness

## Abstract

Low-alloy structural steels subjected to quenching and tempering to achieve high strength possess a common drawback associated with low-impact toughness at low temperatures. An additional warm rolling, i.e., tempforming, is a promising approach to strengthen the rolled semi-products along with increasing their impact toughness. The effect of tempforming at 823–923 K on the microstructures and the mechanical properties of a low-alloy steel was studied in comparison with ordinary tempering at the same temperatures. The tempformed microstructures consisted of highly flattened grains with a transverse grain size of 245 nm to 360 nm depending on tempering temperature. A decrease in the transverse grain size with a decreasing temperature was accompanied by an increase in the total dislocation density (including sub-boundary dislocations) from 3.3 × 10^15^ m^−2^ to 5.9 × 10^15^ m^−2^. The steel samples subjected to tempforming exhibited enhanced mechanical properties. The yield strength increased by more than 300 MPa, approaching about 1200–1500 MPa depending on tempforming temperature. Moreover, strengthening by tempforming was accompanied by an increase in the impact toughness, especially inthe low temperature range down to 77 K, where the impact toughness was above 80 J cm^−2^.

## 1. Introduction

Carbon and low-alloy steels (i.e., containing no more than 4% alloying elements) are the most widely used structural materials [1,2,3]. The good mechanical performance after appropriate thermomechanical treatment and reasonable price make these materials highly attractive for various engineering applications. A common disadvantage of carbon and low-alloy steels is their rather high temperature of ductile-to-brittle transition, below which the impact toughness of the steels becomes quite low, making the steels unsuitable as structural materials serving at lowered temperatures [4], e.g., for construction fasteners in the Far North regions. Generally, the impact toughness can be enhanced by thermomechanical treatment that softens the steel. As a rule, softer steels are characterized by higher impact toughness [4]. On the other hand, softening is not frequently desirable for structural steels and alloys. Therefore, various thermomechanical treatments that increase impact toughness without a remarkable decrease in strength are of great practical importance.

Ausforming and its modified version were elaborated to improve the impact toughness of high-strength alloy steels [5]. These treatments include warm working of metastable austenite followed by martensitic transformation. Insufficient austenite stability under conditions of warm working, however, hampers the usage of ausforming for carbon and low-alloy steels. A promising approach to increase the impact toughness of carbon steels was developed by Kimura et al. [6]. This approach is based on tempforming, i.e., large strain warm rolling following tempering at the same temperature. Interestingly, tempforming increases the impact toughness along with significantly strengthening the steels. Moreover, a substantial increase in the impact toughness occurs at lowered temperatures. The steels subjected to tempforming occasionally demonstrate a unique phenomenon that is inverse temperature dependence of their impact toughness [7]. The high impact toughness of the tempformed steels was attributed to delamination of specimens across the crack propagation that is associated with the warm-rolled microstructure consisting of highly elongated grains. However, the structural mechanisms of such delamination toughness are still unclear. The effect of tempforming conditions on the delamination mechanisms has not been clarified. Previous studies have revealed the importance of large strains during tempforming on the delamination toughness at lowered temperatures [5,8], whereas the tempforming temperature effect on the impact toughness behavior deserves further investigation.

The high strength of the tempformed steels results from the small transverse grain size, the high dislocation density, and the finely dispersed carbides [9]. Though the mentioned structural strengthening mechanisms are rather well-elaborated [10], their fractional contributions to the overall yield strength of warm-worked steels are still a subject of some debate [9,11]. Takaki et al. pointed out the dislocation strengthening being solely responsible for the yield strength of work-hardened steels [11]. On the other hand, various structural strengthening mechanisms were frequently discussed as mutually independent and linearly additive [10,12]. The uncertainty in the strength calculation/prediction is associated with a lack of adequate experimental results to select the correct approach. The present study is aimed to make up for a deficiency in the experimental investigations of work-hardened microstructures and to highlight the structure–property relations in a typical representative of widely used low-alloy structural steels subjected to tempering followed by warm rolling.

## 2. Materials and Methods

**Figure 1:**
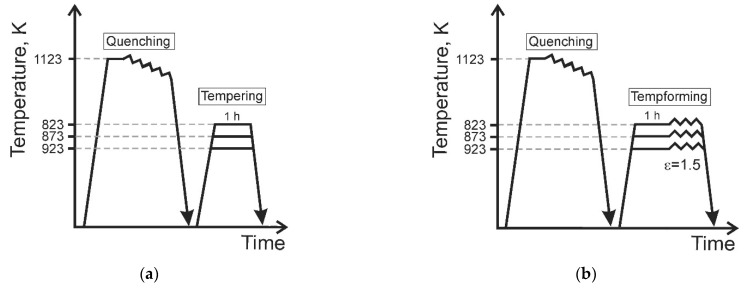
Schematic drawings of tempering (**a**) and tempforming (**b**) used in the present study for steel processing.

A low-alloy steel of type AISI 4135 with a chemical composition of Fe-0.36C-0.4Si-0.56Cr-0.57Mn-0.54Mo-0.0067P-0.0034S (all in mass%) was hot rolled at 1123 K followed by water quenching. Two sets of steel samples were prepared to reveal some advantages of tempforming over common tempering (Figure 1). The quenched steel samples were tempered for 1 h at 823 K, 873 K, or 923 K (Figure 1a). Then, some samples tempered at different temperatures were subjected to plate rolling at tempering temperature to a total strain of 1.5 (Figure 1b).

The structural observations were performed on the RD-ND sections (RD is the rolling direction, and ND is the normal direction) using a Quanta 600 FEG scanning electron microscope (SEM) equipped with an electron backscattering diffraction (EBSD) pattern analyzer incorporating an orientation imaging microscopy (OIM) system. The fine structures were studied with a JEOL JEM-2100 transmission electron microscope (TEM) operating at 200 kV. The specimens for structural investigations were electropolished using an electrolyte containing 10% perchloric acid and 90% acetic acid at a voltage of 20 V at room temperature. The OIM images were subjected to cleanup procedure setting 5 as a minimal number of points per grain with a tolerance misorientation angle of 5°. The mean transverse grain size was evaluated on the OIM images as the average distance between high-angle boundaries with misorientations of *θ* ≥ 15° along ND. An average misorientation of low-angle sub-boundaries was evaluated in the misorientation range of 2° ≤ *θ* < 15° by using the OIM software. The mean subgrain size was evaluated by the line intercept method counting about 100 subgrains on at least 5 typical TEM micrographs. The dislocation densities in the grain/subgrain interiors were evaluated counting the individual dislocations on at least 6 representative TEM images. The average size of the dispersed particles was measured on TEM micrographs counting more than 30 particles for each data point. The volume fractions of second-phase particles at various temperatures were calculated by Thermo-Calc software using TCFE-7 database.

The tensile tests were carried out at an ambient temperature at a crosshead rate of 2 mm/min by using an Instron 5882 testing machine on specimens with a gauge length of 12 mm and a cross section of 3 mm × 1.5 mm (British Standards: BS 18). The tension direction was parallel to the rolling direction. Standard (ASTM E 23) Charpy V-notch specimens were tested using an Instron 450 J impact machine with an Instron Dynatup Impulse data acquisition system at temperatures ranging from 77 K to 293 K. The specimens for impact tests were cut from the rolled plates so that the impact direction was parallel to the normal direction. At least two specimens were tested for each datapoint of strength and impact toughness.

## 3. Results

### 3.1. Developed Microstructures

**Figure 2:**
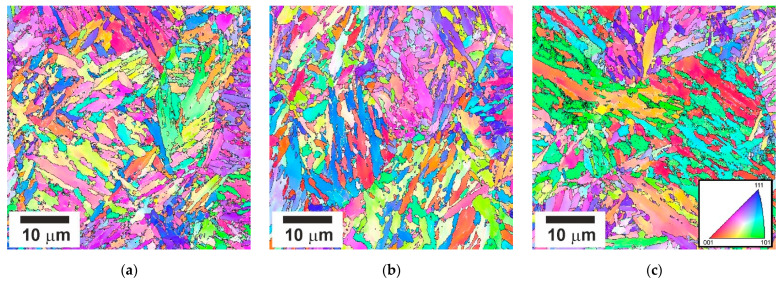
Typical microstructures in a low-alloy steel after tempering at 823 K (**a**), 873 K (**b**), and 923 K (**c**).

**Figure 3:**
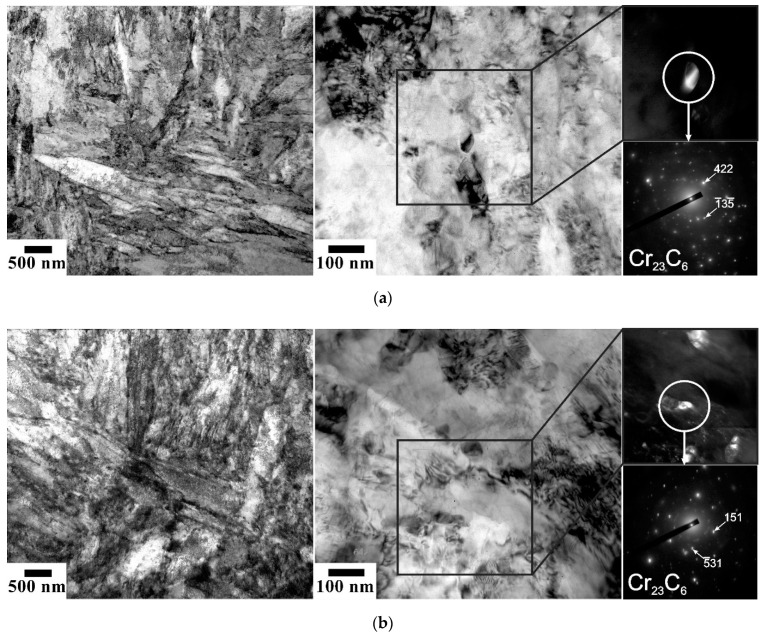

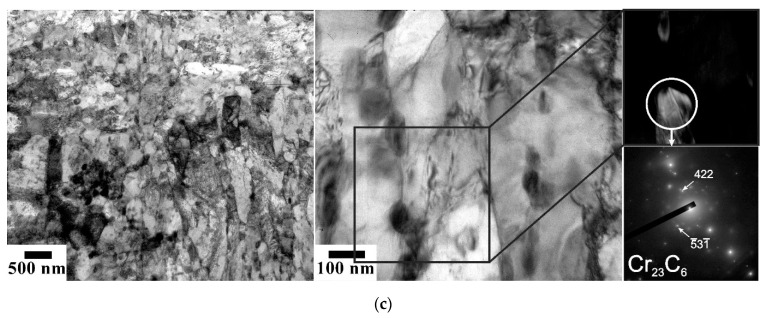
TEM images of the fine structures evolved in a low-alloy steel after tempering at 823 K (**a**), 873 K (**b**), and 923 K (**c**). The dark field images were obtained using the indicated diffraction spots from carbide particles.

Tempering at 823–923 K for 1 h results in the evolution of tempered martensite laths with a relatively high dislocation density and numerous dispersed particles. Typical tempered microstructures are shown in Figure 2. The size of the martensite structural elements, i.e., packets, blocks, and laths, does not remarkably depend on the tempering temperature in the studied range of 823–923 K. The high-angle boundary spacing is about 800 nm. Figure 3 displays a series of representative TEM images of fine structures in the samples subjected to tempering. An average lath boundary spacing slightly increases from 190 nm to 220 nm as the tempering temperature increases from 823 K to 923 K. The tempered lath martensite contains numerous dispersed particles, mainly Cr_23_C_6_ precipitates, with an average size increasing slightly from 40 nm to 50 nm with tempering temperature. The overwhelming majority of Cr_23_C_6_ particles were attributed to the superior precipitation kinetics of Cr_23_C_6_ carbide among other possible precipitations in such low-alloy steels [9].

**Figure 4:**
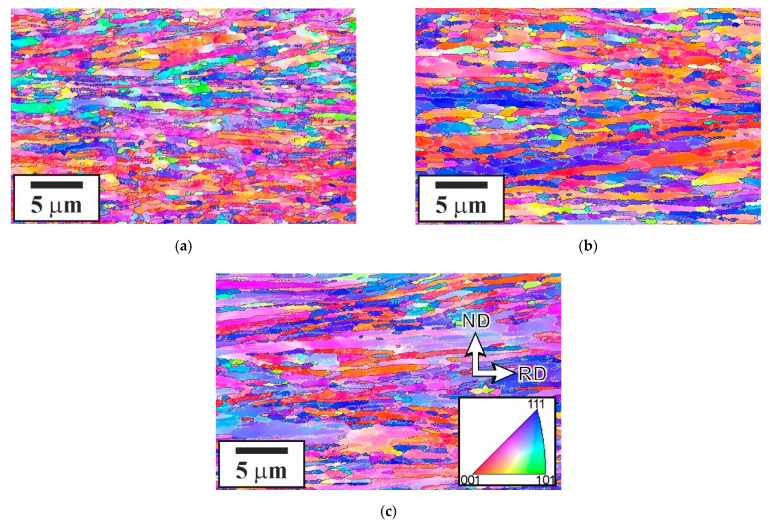
Typical microstructures in a low-alloy steel subjected to tempforming at 823 K (**a**), 873 K (**b**), and 923 K (**c**). The colors indicate crystallographic direction along ND.

**Figure 5:**
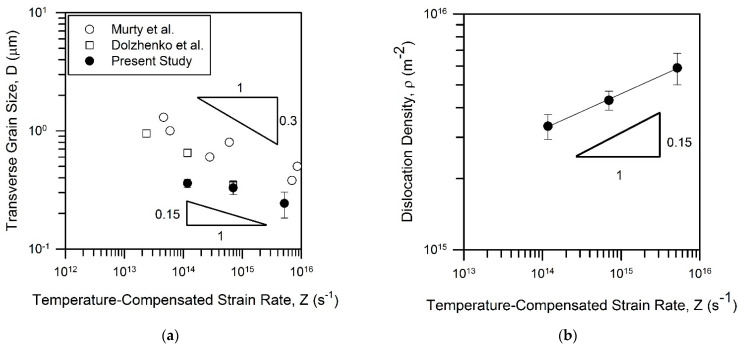
Effect of temperature-compensated strain rate during warm working on the transverse grain size (**a**) and the dislocation density (**b**) in low-alloy steels. The reference data were adapted from [9,14].

The representative microstructures evolved in the steel samples subjected to tempforming at 823–923 K are shown in Figure 4, and the microstructural parameters are listed in Table 1. The tempformed microstructures are characterized by ultrafine grains that are highly elongated along the rolling direction. The transverse grain size decreases from 360 nm to 245 nm with tempforming temperature. Taking the activation energy as that for self-diffusion in low-alloy steels [13], the effect of the temperature-compensated strain rate (*Z*) on the transverse grain size (*D*) is represented in Figure 5a along with some literature data [9,14]. The present data can be roughly expressed by a power law function of *D*~*Z*^−0.15^. It should be noted that except for tempforming at 923 K, the present results obey a powerlaw function of *Z* with an exponent of about −0.3 much similar to other studies of warm to hot deformation of low-alloy steels. The much finer transverse grain size in the sample subjected to tempforming at 923 K (*Z* = 2 × 10^14^ s^−1^ in Figure 5a) than could be expected by the extrapolation of the *D–Z* relationship from the range of high strain rates can be explained by a large fraction of the second-phase particles in the present steel.

The dislocation density in the low-angle subgrain boundaries can be evaluated as follows [12]:

*ρ*_B_ = 1.5*S*v*θ*/*b* (1)

Here, *S*v is the surface area of low-angle sub-boundaries per unit volume; *θ* is an average misorientation of these sub-boundaries, and *b* is the Burgers vector. Adding the dislocation density in the grain/subgrain interiors (*ρ*_IN_), the effect of processing conditions on the total dislocation density (*ρ* = *ρ*_B_ + *ρ*_IN_) is shown in Figure 5b. The dislocation density can be expressed by a powerlaw function of the temperature-compensated strain rate with an exponent of 0.15. The latter roughly corresponds to the absolute value of the exponent in the *D–Z* relationship for the whole strain range in the present study. In turn, the inverse dependence, i.e., *D*~1/*ρ*, corresponds to the common viewpoint on the microstructural and dislocation strengthening [15,16,17].

**Figure 6:**
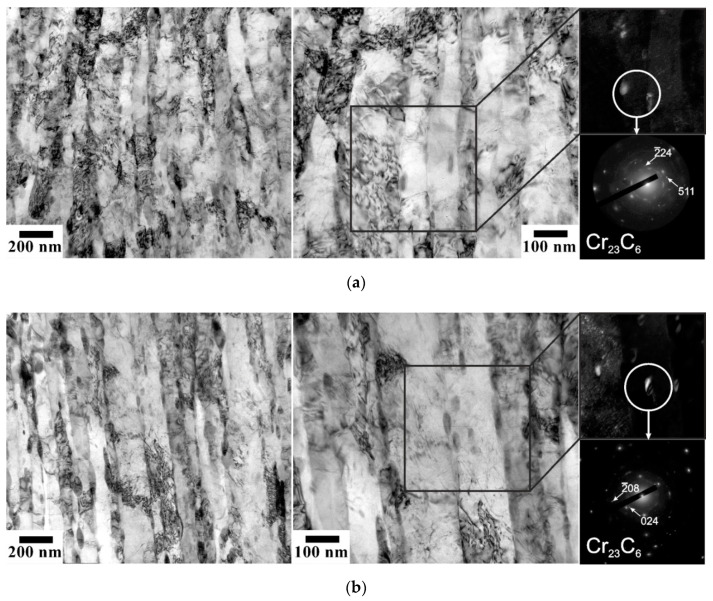

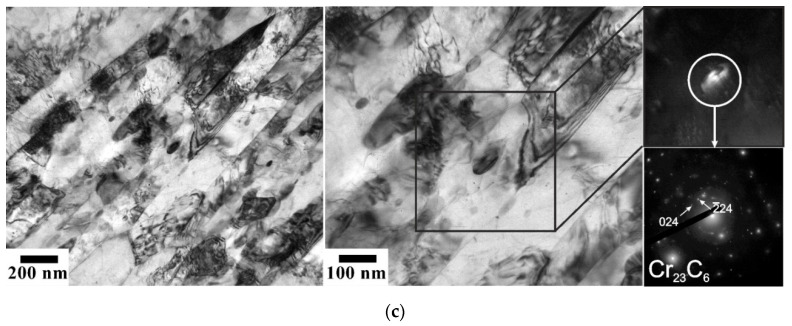
Typical TEM images of the fine structures in a low-alloy steel after tempforming at 823 K (**a**), 873 K (**b**), and 923 K (**c**). The dark field images were obtained using the indicated diffraction spots from carbide particles.

**Figure 7:**
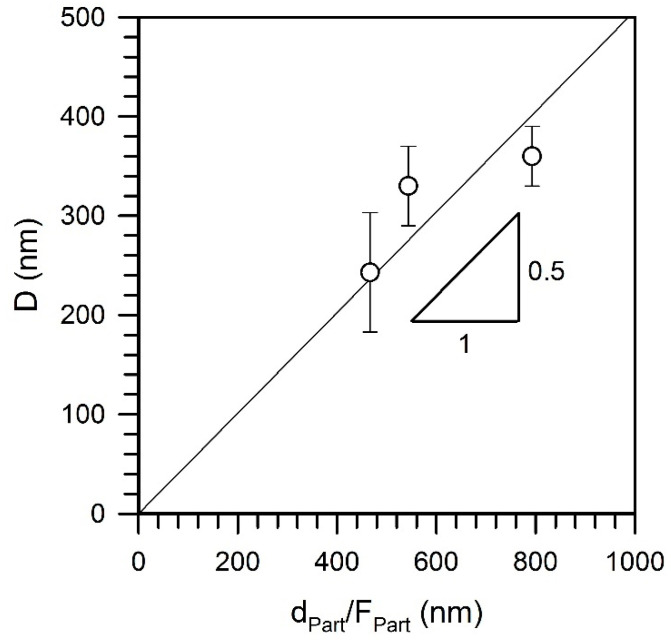
Relationship between the transverse grain size (*D*) and a ratio of the particle size (*d*_Part_) to the particle volume fraction (*F*_Part_) in a low-alloy steel subjected to tempforming.

Tempforming results in the evolution of lamellar-type substructures containing highly elongated subgrains with a transverse size of 80 nm to 150 nm depending on the tempforming temperature similar to the tempformed microstructures (Figure 6). In addition, the size of the second-phase particles changes during tempforming, especially at the lowest temperature of 823 K (Table 1). It should be noted that the transverse grain size correlates with the size of the second-phase particles. Figure 7 shows that the grain size increases as the particle size increases or the particle volume fraction decreases. The slope of a linear approximation passing through the origin in Figure 7 is 0.5, which is close to that of 2/3 as originally suggested by Zener for the limiting grain size [18,19].

**Figure 8:**
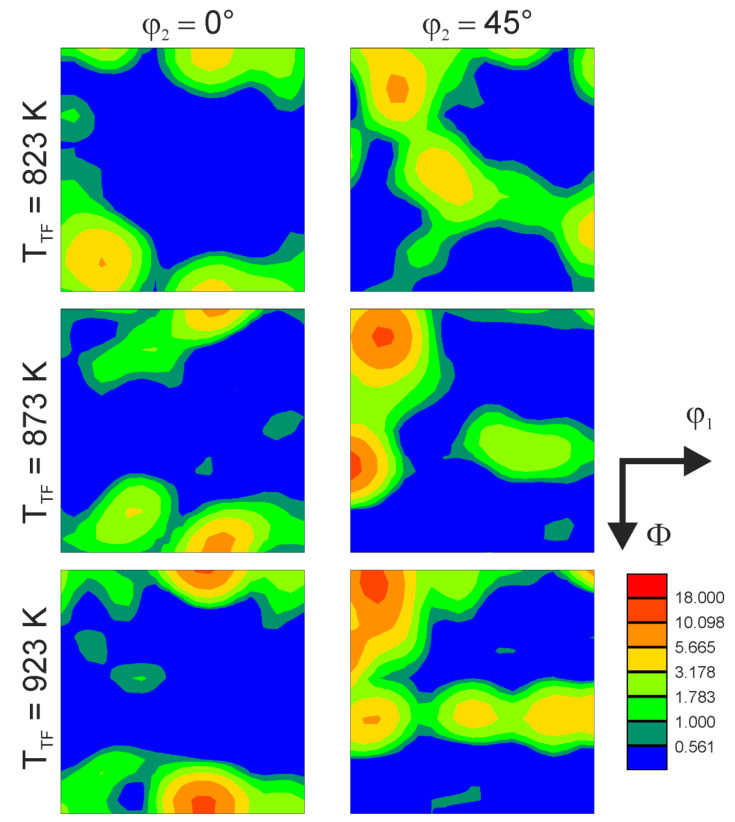
Sections of ODFsat φ_2_ of 0° and 45° for a low-alloy steel subjected to tempforming at indicated temperatures.

The textures that develop by tempforming are shown in Figure 8 as sections of orientation distribution functions (ODFs) at φ_2_ of 0° and 45°. A rather strong texture component of rotated cube, {001}<110>, develops in the tempformed samples. In addition to a rotated cube, the tempformed samples are characterized by the γ-fiber, <111>//ND, and α-fiber, <110>//RD. The intensity of the texture including the rotated cube and the fibers apparently increases with increasing temperature. Remarkable texture components of {111}<110> and {223}<110> develop during tempforming at 873 K and 923 K. Such textures have been frequently observed in bcc metals and alloys subjected to plate rolling with large reductions in thickness [20,21]. The rotated cube texture has been considered responsible for easy delamination of rolled steel plates crosswise to the ND during impact tests at lowered temperatures [5].

### 3.2. Mechanical Properties

#### 3.2.1. Tensile Tests

**Figure 9:**
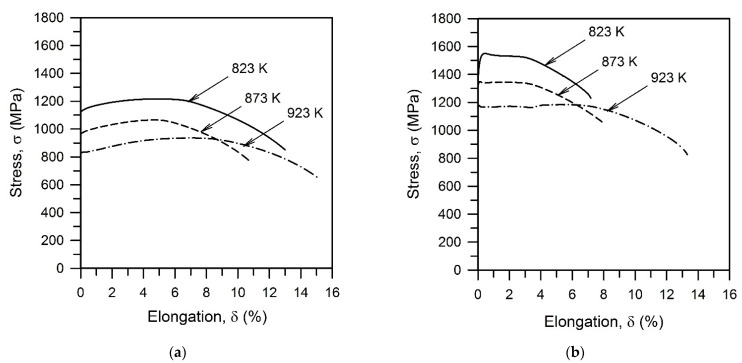
Tensile stress–elongation curves obtained during tensile tests at room temperature of a low-alloy steel subjected to tempering (**a**) and tempforming (**b**) at indicated temperatures.

The effect of tempforming on the tensile behavior at ambient temperature of the tempered steel samples is illustrated by Figure 9. The tensile behavior of the tempered samples is characterized by a small strain hardening in the range of elongation up to about 5% irrespective of tempering temperature (Figure 9a). Following the yielding, the flow stresses progressively increase to a maximum and then gradually decrease due to necking until failure. The shape of the stress–strain curve does not depend significantly on tempering temperature. A decrease in tempering temperature by 50 K increases the flow stress by about 135 MPa; i.e., the flow curve shifts as a whole toward the higher stress as tempering temperature decreases. A decrease in the strength with increasing tempering temperature is accompanied by a little increase in plasticity.

**Figure 10:**
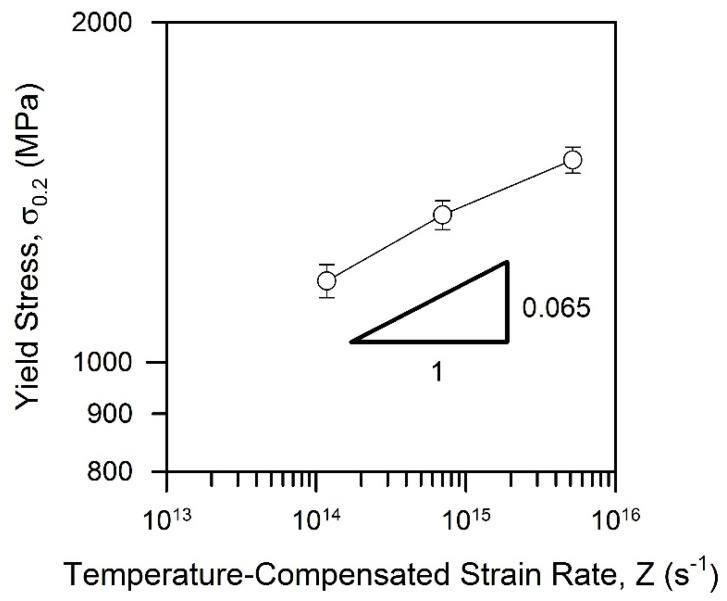
Effect of temperature-compensated strain rate during tempforming on the yield strength of a low-alloy steel.

Tempforming remarkably increases the yield strength of the tempered steel samples by about 400 MPa (Figure 9b). The strengthening effect of tempforming temperature also increases. Each change in tempforming temperature by 50 K in the studied range of 823–923 K changes the yield strength by about 165 MPa. On the other hand, the strain hardening in the elongation range up to 4% decreases to almost zero. In contrast to the tempered steel samples, therefore, remarkable steady-state-like deformation behavior followed by necking and failure took place during the tensile tests of the tempformed ones. The effect of tempforming conditions on the yield strength of the processed steel samples can be represented by a power law function of the temperature-compensated strain rate, *Z*, (Figure 10). An exponent of 0.065 in Figure 10 is about half of that in the *ρ–Z* relationship in Figure 5b that suggests the dislocation strengthening as the dominant strengthening mechanism for the tempformed steel samples.

#### 3.2.2. Impact Toughness

**Figure 11:**
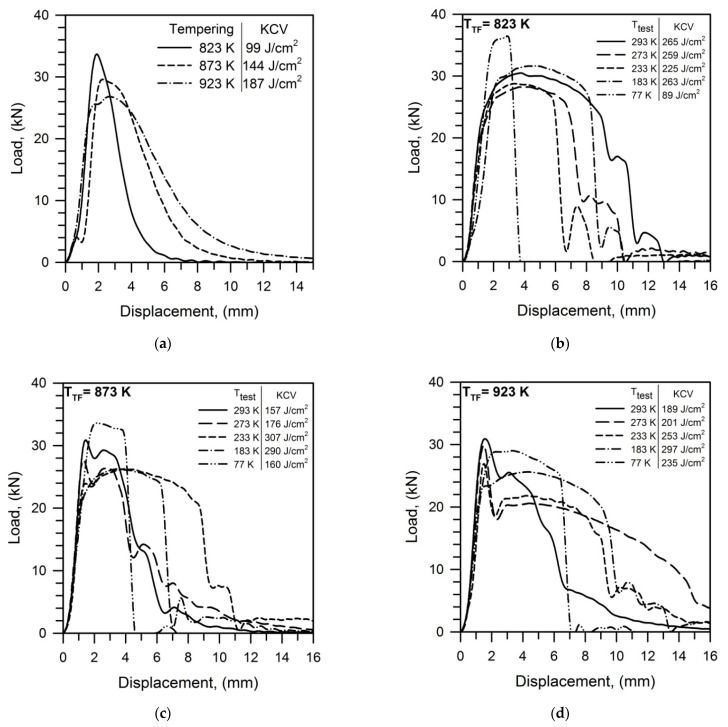
Impact load-displacement curves obtained for a low-alloy steel subjected to tempering at indicated temperatures (**a**) and tempforming at 823 K (**b**), 873 K (**c**), or 923 K (**d**). T_test_ indicates the impact test temperature for the tempformed steel samples.

The effect of tempforming on the impact toughness of tempered steel samples is shown in Figure 11 as a series of the load-displacement curves recorded at various temperatures of the impact tests. The impact curves of the samples after conventional tempering look like typical ones with a general yield close to a maximum load followed by a rapidly decreasing load due to cracking and fracture (Figure 11a). An increase in tempering temperature decreases both the general yield and the maximum load, while the plastic stage before cracking increases and the rate of crack propagation decreases that promotes the impact toughness. Thus, the impact toughness comprises 100–200 J cm^−2^ depending on tempering temperature.

In contrast, the steel samples subjected to tempforming possess much higher impact toughness. The load-displacement curves for the tempformed steel samples are characterized by pronounced stages of sluggish fracture following maximum load. A remarkable stage of plastic deformation between the general yield and the maximum load takes place in the samples tempformed at 823 K, leading to the impact toughness well above 200 J cm^−2^ even at a testing temperature lowered to 183 K (Figure 11b). The steel samples subjected to tempforming at 873–923 K exhibit sharp peaks on the impact curves at small displacements followed by a load increase to another maximum with a subsequent slow fracture (Figure 11c,d). Such a substantial expansion of the deformation stage during impact tests provides high impact toughness above 150 J cm^−2^ even at cryogenic temperature.

**Figure 12:**
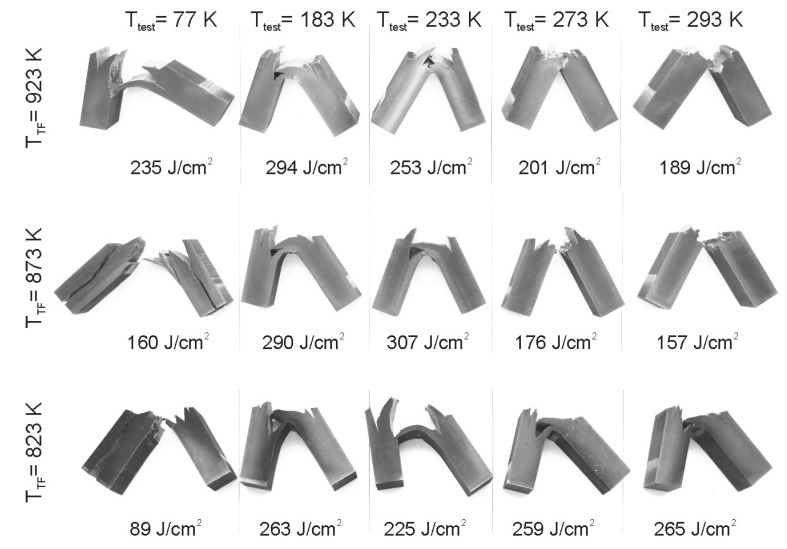
Impact specimens and impact toughness after tests at indicated temperatures (*T*_test_) of a low-alloy steel subjected to tempforming at different temperatures (*T*_TF_).

**Figure 13:**
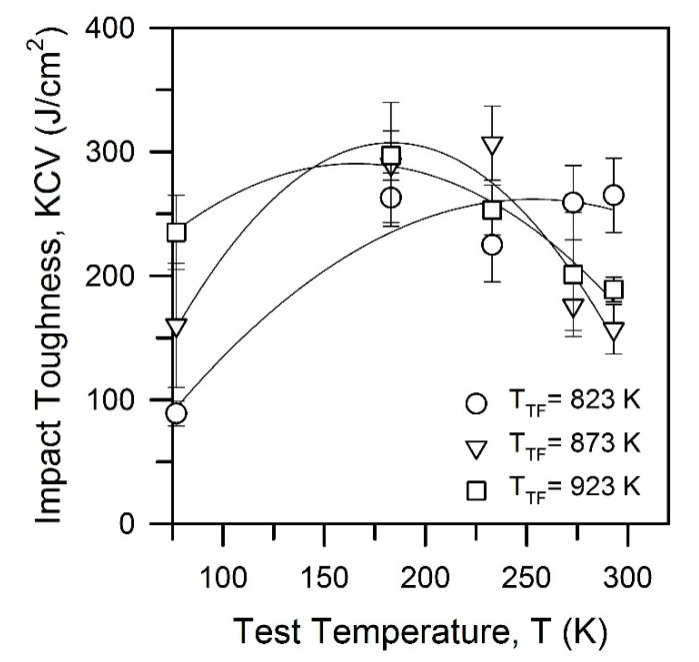
Effects of tempforming temperature and test temperature on the impact toughness of a low-alloy steel.

**Figure 14:**
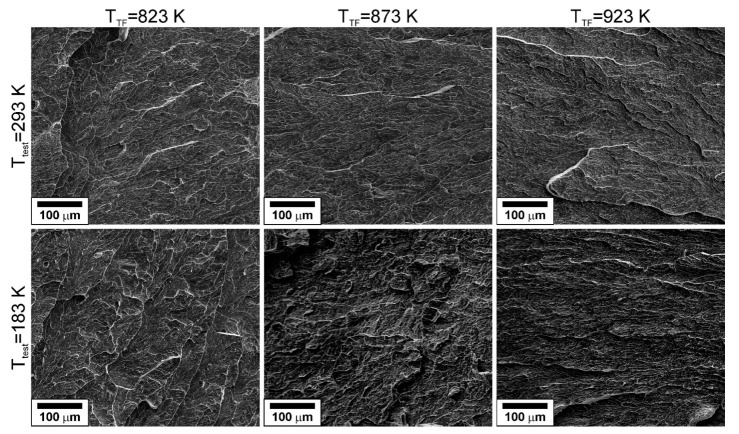
Fracture surfaces of specimens made of a low-alloy steel subjected to tempforming at indicated temperatures (*T*_TF_) and tested at temperatures (*T*_test_) of 183 K or 293 K.

Figure 12 presents the specimens after impact tests. It is clearly seen in Figure 12 that the specimens experience distinct delamination during the tests. It is worth noting that the impact specimens do not fracture completely on the impact tests at lowered temperatures of 213–233 K. The delaminated specimens bend and pass through the specimen holder of the testing machine. Thus, the impact toughness surprisingly increases with a decrease in test temperature below 293 K. Variations of the impact toughness with test temperature are represented in Figure 13, where quadratic polynomial regression fits were used for the experimental data to facilitate comparison. The impact toughness increases to its maximum and then decreases as the test temperature decreases in the range of 77–293 K. The steel samples tempformed at 823 K are characterized by the highest impact toughness at 293 K, whereas their impact toughness is the lowest among the other samples at 77 K. In contrast, the steel samples tempformed at 923 K exhibit an opposite tendency, i.e., relatively low and high impact toughness at room and cryogenic temperatures, respectively. An increase in tempforming temperature leads to an apparent shift of the maximum impact toughness in Figure 13 toward low temperatures. Representative examples of the fracture surfaces of the specimens after impact tests at various temperatures are shown in Figure 14. The brittle fracture surfaces with wavy lines and ridges crossing the cleavage terraces in Figure 14 suggest the transgranular fracture mode for delamination of all specimens. The terrace size does not depend remarkably on tempering temperature or test temperature, except that when tempered at 923 K and tested at room temperature,it exhibits apparently the finest ridge patterns with the largest steps between the cleavage terraces.

## 4. Discussion

Tempforming allows us to significantly improve the mechanical properties of low-alloy steels. It should be noted that both the tensile strength and the impact toughness are improved highlighting the special benefit of tempforming. Kimura et al. in early studies discussed the ultrafine-grained microstructure with finely dispersed carbides as the main strengthening contributors of the carbon steels after tempforming [6,7,22]. On the other hand, the dislocation strengthening (or work hardening) is generally considered as a strengthening factor for various steels and alloys subjected to large strains by cold or warm plastic deformations [23,24,25]. Therefore, there could be three strengthening mechanisms that deserve detailed consideration for low-alloy steels subjected to tempforming. Those are the grain size strengthening [26,27],

Δσ_D_ = *k*_y_*D*^−0.5^, (2)

the dispersion strengthening [28]

Δσ_Or_ = 0.55 *Gb λ*^−1^ (ln(0.5*d*_P_*/*b*) + 0.7), (3)

or [29]

Δσ_G_ = 0.0165 (Δ*G*)^3/2^ (2*F*_P_/*G*)^0.5^ (0.5*d*_F_/*b*)^0.275^, (4)

and the dislocation strengthening [23],

Δσ_ρ_ = α*Gb*
*ρ*^0.5^, (5)

where *k*_y_ is the grain size strengthening factor of about 240 MPa μm^0.5^ for low-carbon steels [29,30]; D is the mean grain size; *G* = 81,000 MPa is the shear modulus [13]; *b* = 0.25 nm is the Burgers vector [13]; *λ* is the edge-to-edge particle spacing;d_P_* depends on the mean particle size (*d*_P_) and can be calculated as *d*_P_* = (*d*_P_^−1^ + *λ*^−1^)^−1^; Δ*G* is the difference in shear modulus between precipitate and matrix (about 66 GPa for carbide particles [31]); *F*_P_ is the particle volume fraction;α is a constant of about 0.9 [11], and ρ is the dislocation density. Note here that the dispersed strengthening is commonly modeled by Orowan looping (Equation (3)) or modulus mismatch (Equation (4)). The mechanism providing smaller strengthening should be considered as the actual one. Modulus strengthening by Equation (4) results in a strength well above 1000 MPa. Thus, the Orowan mechanism (Equation (3)) will be considered below.

**Figure 15:**
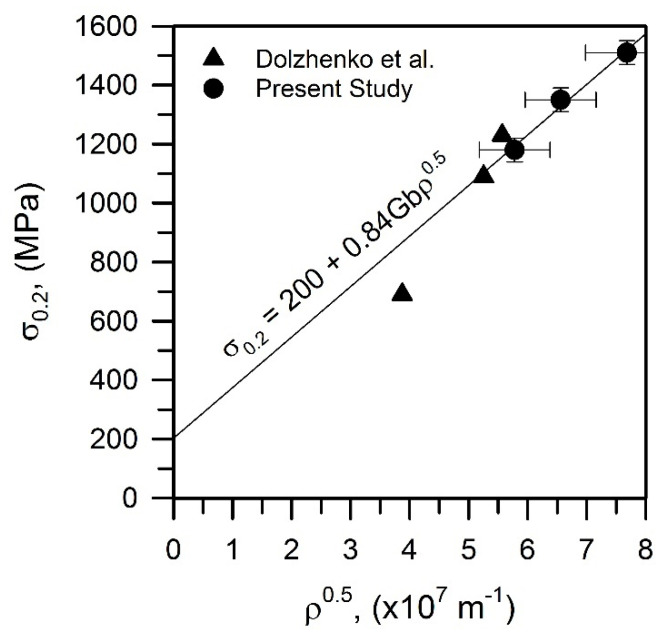
Relationship between the yield strength (*σ*_0.2_) and the dislocation density (*ρ*) for the present steel and another high-strength low-alloy steel [9] subjected to tempforming.

The relationship between the yield strength and the dislocation density (*ρ* in Table 1) is represented in Figure 15. The data of a previous study on a high-strength low-alloy steel subjected to tempforming under similar conditions are also shown in Figure 15 for reference. The yield strength of the present tempformed steel samples, including published data [9] for the similar strength/dislocation density range, can be expressed by the dislocation strengthening with a strengthening factor of 0.84, which is quite close to that of 0.9 reported for low-alloy steels [11].

**Figure 16:**
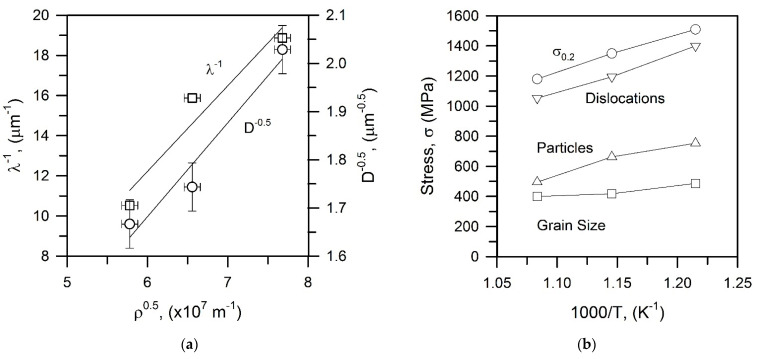
Relationships among the dislocation density, *ρ*, the interparticle spacing, *λ,* and the transverse grain size, *D* (**a**) and a comparison of the dislocation, particle, and grain size strengthening with experimental yield strength, *σ*_0.2_, (**b**) for a low-alloy steel subjected to tempforming.

The dislocation density and the grain size depend on the tempforming conditions expressed by the temperature-compensated strain rate (Figure 5). In addition, the grain size depends on the particle dispersion (Figure 7). Specific power law functions revealed between the microstructural parameters and processing conditions, i.e., *D*~*Z*^−0.15^ and *ρ*~*Z*^0.15^, suggest a linear relationship between these strengthening mechanisms (*D*^−0.5^~*ρ*^0.5^). It is clearly seen in Figure 16a that the dispersion strengthening and the grain size strengthening can also be related to the dislocation strengthening through linear functions. Takaki et al. suggested that the yield strength of work-hardened steels can be expressed by using the dislocation density as a unique strengthening parameter [11]. It should be noted here that the grain size strengthening (Equation (2)) and the dispersion strengthening (Equation (3)) relate to the microstructural parameters with the dislocation motion, which in turn determine the flow stress [26,27,28]. Thus, an increase in the dislocation density (work hardening) should depend on the microstructure and the processing conditions. The strengthening from different contributors as calculated by using Equations (2), (3) and (5) is represented in Figure 16b in comparison with the experimental yield strength (*σ*_0.2_). It is interesting that the summation of the grain size strengthening and the dispersion strengthening results in the strength, which is almost the same as that from the dislocation strengthening. The present results are thereforein line with previous discussions [9,11] that the yield strength can be solely expressed by the dislocation density evolved in the steel samples subjected to tempforming, i.e., large strain warm rolling.

**Figure 17:**
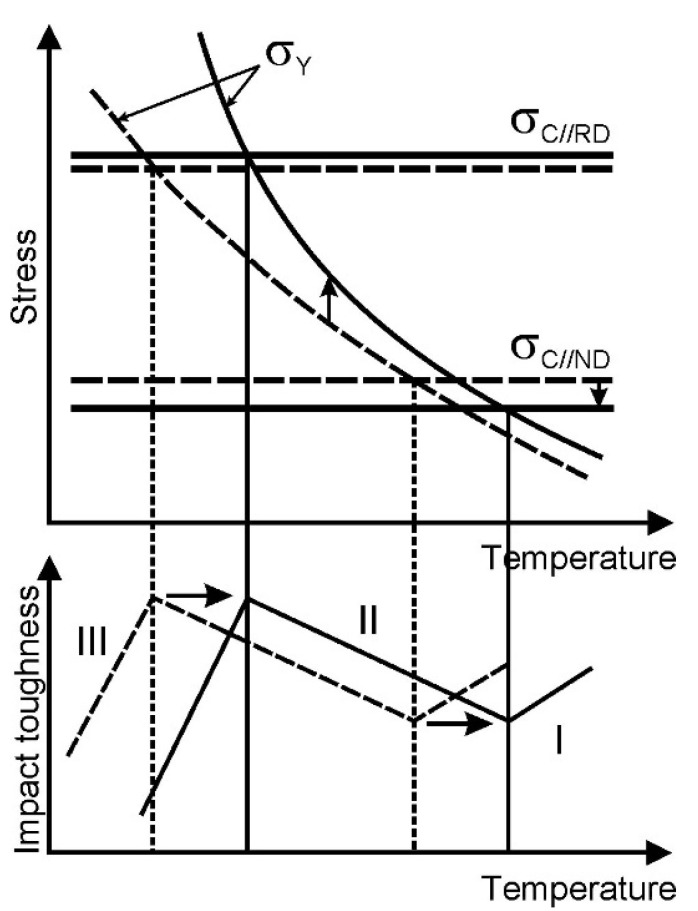
Modified Yoffee diagram illustrating the effect of tempforming temperature on the fracture behavior of a low-alloy steel at low temperatures.

The delamination of specimens across the primary crack propagating during impact tests has been considered as the principal mechanism improving the impact toughness of the tempformed steel samples [7,22]. The paradox of the delamination toughness is that the brittle fracture favors the impact toughness. The rapid cleavage crosswisein the impact direction blunts the notch, releases the stress concentration, and branches the main crack. The readier the cleavage is along the specimen, the higher the impact toughness that can be obtained. The delamination toughness is illustrated by the modified Yoffee diagram (Figure 17) [32,33]. The coherence length of {001} cleavage planes in the tempformed steels is maximal in the rolling direction and minimal in the transverse direction owing to strong rotated cube texture in highly flattened grains. Therefore, the cleavage fracture stress along the normal direction (*σ*_C_//ND in Figure 17) is minimal, while that along the rolling direction (*σ*_C_//RD) is maximal irrespective of temperature. In contrast, the effective yield stress (*σ*_Y_) increases with a decrease in temperature [4]. Thus, the delamination toughness provides an increasein the impact toughness and a decrease in temperature in the range of *σ*_C_//RD > *σ*_Y_ > *σ*_C_//ND (region II in Figure 17).

The large steps between the cleavage terraces on the fracture surface of the specimen made of steel sample tempformed at 923 K and tested at room temperature (s. Figure 14) are indicative of a relatively high *σ*_C_//ND. As a result, this specimen exhibits rather low-impact toughness at 293 K. On the other hand, a decrease in tempforming temperature results in a decrease in *σ*_C_//ND, while the *σ*_C_//RD remains almost unchanged, and *σ*_t_ increases significantly, especially at low temperatures owing to substantial work hardening (dislocation strengthening). Hence, the peak of the impact toughness at low temperatures (the border between regions II and III in Figure 17) tends to appear at higher temperatures. An increase in tempforming temperature is beneficial for impact toughness at cryogenic temperatures, whereas low tempforming temperature improves the impact toughness at room and lowered temperatures. A similar effect on the low-temperature impact toughness of tempformed steel samples has been recently achieved by an additional annealing at a somewhat elevated temperature after tempforming [34].

Tempforming provides a unique combination of high strength and high impact toughness, especially at low temperatures in some low-alloy and plain carbon structural steels [9,22]. Such surprising properties of processed steels result from specific lamellar-type microstructures evolved during large strain warm rolling. Further improvement of strength and ductility can be achieved by a refinement of the original microstructure [35]. The mechanical performance of the tempformed steels thus depends on the microstructural parameters. The relationships between the developed microstructures and the tempforming conditions revealed in the present study can be used to adapt tempforming to other structural steels. Given the chemical and phase contents of a steel, appropriate processing conditions can be selected to obtain the desired microstructure and mechanical properties. The strong grain size and texture anisotropy as observed in the present and previous relevant studies on tempforming suggest remarkable difference in the mechanical properties of processed steels along the major rolling directions that deserve further close investigation.

## 5. Conclusions

The effect of tempforming at 823–923 K on the microstructures and the mechanical properties was studied for a low-alloy steel, Fe-0.36C-0.4Si-0.56Cr-0.57Mn-0.54Mo-0.0067P-0.0034S (all in mass%). The main results can be summarized as follows.

1. The tempformed microstructures consisted of highly flattened grains with high dislocation density. The transverse grain size decreased from 360 nm to 245 nm, while the dislocation density increased from 3.3 × 10^15^ m^−2^ to 5.9 × 10^15^ m^−2^ as the tempforming temperature decreased from 923 K to 823 K. The grain size and the dislocation density could be expressed by power law functions of a temperature-compensated strain rate with exponents of −0.15 and 0.15, respectively. Moreover, the linear function was observed between the grain size and the carbide particle dispersion quite similar to the pinning relationship originally proposed by Zener.
2. The tempformed steel samples were characterized by enhanced mechanical properties. The yield strength increased from 1180 MPa to 1510 MPa with a decrease in tempforming temperature. The strengthening was accompanied by an increase in the impact toughness, which surprisingly increased with a decrease in temperature of the impact test below 293 K. The impact toughness of well above 200 J cm^−2^ was observed at temperatures of 183–233 K. Even at a cryogenic temperature of 77 K, the impact toughness exceeded 80 J cm^−2^.
3. The strengthening of the tempformed steels samples was attributed to the high dislocation density. The yield strength could be related to the dislocation strengthening or additive contributions of the grain size strengthening and the dispersion strengthening. Such an alternative resulted from the linear relationships between the dislocation strengthening and both the grain size strengthening and the dispersion strengthening.
4. The improvement of the impact toughness after tempforming was associated with a delamination of the specimens crosswise to the impact direction because of cleavage fracture, which occurred readily along the specimen. A decrease in tempforming temperature promoted the impact toughness at room and lowered temperatures, whereas tempforming at a relatively high temperature increased the impact toughness at the cryogenic temperature.

## Figures and Tables

**Table 1:** The transverse grain size (*D*), the transverse subgrain size (*d*), the dislocation density (*ρ*), the mean size (*d*_P_), and the volume fraction (*F*_P_) of second-phase particles evolved in a low-alloy steel by tempforming at different temperatures (*T*_TF_).

Parameter	*T*_TF_ = 823 K	*T*_TF_ = 873 K	*T*_TF_ = 923 K
*D* (nm)	245 ± 60	330 ± 40	360 ± 30
*d* (nm)	80 ± 2	100 ± 3	150 ± 7
*ρ* (×10^15^ m^−2^)	5.9 ± 0.9	4.3 ± 0.4	3.3 ± 0.4
*d*_P_ (nm)/*F*_P_	25 ± 3/0.0536	32 ± 3/0.058	53 ± 8/0.0669

## Data Availability

The data presented in this study are available on request from the corresponding author.
